# A novel necroptosis-related gene signature associated with immune landscape for predicting the prognosis of papillary thyroid cancer

**DOI:** 10.3389/fgene.2022.947216

**Published:** 2022-09-15

**Authors:** Zhiyuan Wang, Pu Wu, Jinyuan Shi, Xiaoyu Ji, Liang He, Wenwu Dong, Zhihong Wang, Hao Zhang, Wei Sun

**Affiliations:** Department of Thyroid Surgery, The First Hospital of China Medical University, Shenyang, Liaoning, China

**Keywords:** necroptosis, signature, microenviroment, immune, papillary thyroid cancer, prognosis, TCGA

## Abstract

**Background:** Necroptosis, a type of programmed cell death, has been implicated in a variety of cancer-related biological processes. However, the roles of necroptosis-related genes in thyroid cancer yet remain unknown.

**Methods:** A necroptosis-related gene signature was constructed using the least absolute shrinkage and selection operator (LASSO) regression analysis and Cox regression analysis. The predictive value of the prognostic signature was validated in an internal cohort. Additionally, the single-sample gene set enrichment analysis (ssGSEA) was used to examine the relationships between necroptosis and immune cells, immunological functions, and immune checkpoints. Next, the modeled genes expressions were validated in 96 pairs of clinical tumor and normal tissue samples. Finally, the effects of modeled genes on PTC cells were studied by RNA interference approaches *in vitro*.

**Results:** In this study, the risk signature of seven necroptosis-related genes was created to predict the prognosis of papillary thyroid cancer (PTC) patients, and all patients were divided into high- and low-risk groups. Patients in the high-risk group fared worse in terms of overall survival than those in the low-risk group. The area under the curve (AUC) of the receiving operating characteristic (ROC) curves proved the predictive capability of created signature. The risk score was found to be an independent risk factor for prognosis in multivariate Cox analysis. The low-risk group showed increased immune cell infiltration and immunological activity, implying that they might respond better to immune checkpoint inhibitor medication. Next, GEO database and qRT-PCR in 96 pairs of matched tumorous and non-tumorous tissues were used to validate the expression of the seven modeled genes in PTCs, and the results were compatible with TCGA database. Finally, overexpression of IPMK, KLF9, SPATA2 could significantly inhibit the proliferation, invasion and migration of PTC cells.

**Conclusion:** The created necroptosis associated risk signature has the potential to have prognostic capability in PTC for patient outcome. The findings of this study could pave the way for further research into the link between necroptosis and tumor immunotherapy.

## Background

Thyroid cancer is becoming more common in the world. One key explanation is the improved detection of small papillary thyroid cancers (PTCs) using imaging technologies such as ultrasonography (US) has advanced and become more widely used (Sugitani et al., 2021). Aside from PTC, there are three more kinds of thyroid cancer: follicular thyroid cancer (FTC), medullary thyroid cancer (MTC), and anaplastic thyroid cancer (ATC). PTC is the most frequent kind, accounting for more than 80% of all pathological types (Kitahara and Sosa, 2016; Lim et al., 2017; Ito et al., 2018). Although PTC has an overall excellent prognosis, aggressive PTC variants such as the tall cell (TC) and diffuse sclerosing (DS) variants are on the rise and are associated with more aggressive pathologic characteristics that can lead to a poor prognosis or even recurrence in certain patients (Albareda et al., 1998; Carling et al., 2007; Feng et al., 2018). Therefore, searching for new prognostic markers is crucial for the diagnosis, prognosis, and treatment of PTC patients.

Necroptosis, discovered and described in 2005, is a programmed form of necrotic cell death that is distinct from apoptosis, ferroptosis, and pyroptosis (Degterev et al., 2005). It is a caspase-independent and regulated necrotic cell death mechanism that is mechanistically and morphologically identical to apoptosis and necrosis (Christofferson and Yuan, 2010). Necroptosis is regulated by molecules that are also known to govern apoptosis, but it is dependent on the creation of the necrosome, which consists of receptor-interacting serine/threonine-protein kinase 1 (RIPK1) and RIPK3, which activates the pseudokinase mixed lineage kinase (MLKL). MLKL then mediates the release of intracellular materials, which results in the execution of the necroptotic program (Pasparakis and Vandenabeele, 2015; Gong et al., 2019). Necroptosis is not only crucial in the development and progression of numerous immune system disorders and viral infections, but it also plays an important role in cancer biology regulation, including oncogenesis, cancer metastasis, cancer immunity, and cancer subtypes (Stoll et al., 2017; Seehawer et al., 2018). Some dual effects of necroptosis on cancer have been demonstrated as a combination of apoptosis and necrosis. Several articles have reported that necroptosis can trigger inflammatory responses and reportedly promotes cancer metastasis and immunosuppression as a necrotic cell death modality (Seifert et al., 2016; Strilic et al., 2016). Necroptosis is viewed as a barrier that can limit tumor formation when apoptosis is disrupted. As a result, necroptosis has been described as both a friend and a foe of cancer. The expression of necroptosis factors in cancer, as well as their impact on cancer prognosis, is likewise complex. Multiple studies have discovered that numerous important components in necroptotic signaling pathways are downregulated in a variety of malignancies, implying that cancer cells may avoid necroptosis to survive. RIPK3 is downregulated in a variety of malignancies, including breast cancer, colorectal cancer, and melanoma (Feng et al., 2015; Geserick et al., 2015; Koo et al., 2015). Furthermore, decreased RIPK3 expression has been observed to independently predict lower overall survival in colorectal cancer and breast cancer (Feng et al., 2015; Koo et al., 2015). It has also been observed that RIPK1 expression is downregulated in head and neck squamous cell carcinoma, which is associated with disease progression (McCormick et al., 2016). These findings imply that the necrosis pathway has an anti-cancer effect in cancer. However, it does not appear that necroptotic factors are downregulated in all malignancies. These variables have been identified to be elevated in various malignancies, and their overexpression is positively connected with tumor growth. In glioblastoma, for example, the RIPK1 expression is overexpressed in around 30% of cases (grade IV) which leads to a worse prognosis (Park et al., 2009). A recent study has attempted to discover new necroptosis-related signatures in some cancers. Based on necroptosis-related genes, a predictive signature for pancreatic adenocarcinoma was created (Wu et al., 2022). Necroptosis-related genes also play an essential role in tumor immunity in prostate cancer and can be used to predict the prognosis of prostate adenocarcinoma (Li et al., 2021). In stomach adenocarcinoma, a necroptosis-related prognostic signature and a lncRNA SNHG1/miR-21-5p/TLR4 regulatory axis were discovered (Wang and Liu, 2021). However, the prognostic significance of necroptosis-related genes in PTC remains unknown.

Therefore, this study aimed to construct a novel prognostic signature for PTC by detecting the expression and prognostic value of necroptosis-related genes (NRGs) and evaluation of their prognostic value from many aspects. Additionally, a thorough analysis of the link between the signature and the tumor immune microenvironment (TIME) using an external validation *via* the Gene Expression Omnibus (GEO) database was also conducted. Finally, we performed qRT-PCR and cell functional assays to validate the expression and function of the NRGs.

## Methods

A workflow diagram is shown in Supplementary Figure S1.

### Datasets and preprocessing

The Cancer Genome Atlas (TCGA) database (https://portal.gdc.cancer.gov/) was used to obtain the gene expression profiles of thyroid cancer (THCA) (510 tumor and 58 normal samples). Clinical data from TCGA were also retrieved, including age, gender, clinical stage, and survival. Additionally, the raw data of gene expression profiles (*N* = 197) were extracted, processed, and normalized in batches using the “sva” and “limma” packages in R language (version 4.1.2) from seven GEO series [GSE6004 (*N* = 14), GSE33630 (*N* = 49), GSE3467 (*N* = 9), GSE35570 (*N* = 65), GSE29265 (*N* = 20), GSE60542 (*N* = 33), and GSE3678 (*N* = 7)].

To reduce the statistical bias, the clinical data with missing follow-up or fewer than 30 days of follow-up were eliminated. Finally, 498 tumor samples were received with RNA expression data and clinical data, which were randomly divided into the training and test sets with no significant variations in clinical characteristics (Table 1). Furthermore, 67 NRGs were obtained from previous reviews (Additional Files 1).

**TABLE 1:** The clinical characteristics in training, test, and total sets.

Variables	Group	Training set (*N* = 249)	Test set (*N* = 249)	Total set (*N* = 498)	*p* value
Age(year)	≤60	196 (78.71%)	190 (76.31%)	386 (77.51%)	0.520
	>60	53 (21.29%)	59 (23.69%)	112 (22.49%)	
Gender, n (%)	Male	62 (24.90%)	73 (29.32%)	135 (27.11%)	0.267
	Female	187 (75.10%)	176 (70.68%)	363 (72.89%)	
Stage, n (%)	Stage I-II	164 (65.86%)	167 (67.07%)	331 (66.47%)	0.362
	Stage III-IV	83 (33.33%)	82 (32.93%)	165 (33.13%)	
	Unknown	2 (0.80%)	0 (0.00%)	2 (0.40%)	
T, n (%)	T1-2	143 (57.43%)	161 (64.66%)	304 (61.04%)	0.252
	T3-4	105 (42.17%)	87 (34.94%)	192 (38.55%)	
	Tx/unknown	1 (0.40%)	1 (0.40%)	2 (0.40%)	
M, n (%)	M0	152 (61.04%)	130 (52.21%)	282 (56.63%)	0.112
	M1	5 (2.01%)	4 (1.61%)	9 (1.81%)	
	Mx/unknown	92 (36.95%)	115 (46.18%)	207 (41.57%)	
N, n (%)	N0	105 (42.17%)	123 (49.40%)	228 (45.78%)	0.206
	N1	115 (46.18%)	105 (42.17%)	220 (44.18%)	
	Nx/unknown	29 (11.65%)	21 (8.43%)	50 (10.04%)	

### Differential gene expression analysis

The “limma” package in R was employed to identify differentially expressed genes (DEGs) in all tumor and adjacent normal tissues, based on FDR < 0.05.

### Construction of the protein-protein interaction network and functional enrichment of DEGs

A protein-protein interaction (PPI) network was constructed to investigate the DEG interactions using the Search Tool for the Retrieval of Interacting Genes/Proteins (STRING) database (http://www.string-db.org/). For PPI study, the minimum required interaction score was set at 0.9 (The highest confidence). Then, Gene Ontology (GO) and Kyoto Encyclopedia of Genes and Genomes (KEGG) analysis using Metascape (https://metascape.org), a comprehensive tool for gene functional analysis was conducted.

### Consensus clustering based on DEGs

Tumor samples were clustered into distinct subgroups based on necroptosis-related DEGs using R software “ConsensusClusterPlus.” To investigate the overall survival of subgroups, the “survival” and “survminer” packages were employed. The “limma” and “heatmap” packages were used to create a heatmap illustrating the differential expression and association between NRGs and clinical characteristics within each subgroup.

### Construction of necroptosis-related prognostic signature

A training set to identify prognostic DEGs was used to create a risk signature for prognosis. The test set and total set were utilized to validate the predictive potential of created signature. Using the “glmnet” package, the prognostic significance of each DEG was determined using univariate Cox regression analysis. To avoid omissions, we established a cut-off value *p* < 0.2. To avoid model overfitting, the least absolute shrinkage and selection operator (LASSO) penalized Cox proportional hazards regression to identify additional important prognostic genes for overall survival (OS) was used. Then, the detected genes were combined into a multivariate Cox regression model, and the risk scores for each sample were generated using the following formula: risk score = esum (each necroptosis-related gene expression level × corresponding coefficient). Samples were categorized into high- and low-risk categories based on the computed median risk score. To assess predictive capacity, Kaplan–Meier survival curves were examined using the “survival” and “survminer” packages, and receiver operating characteristic (ROC) curves were created using the “timeROC” package. The “stats” package was used to perform principal component analysis (PCA). Finally, univariate and multivariate Cox regression analysis was done to determine whether the risk classification derived from the risk signature is an independent prognostic factor.

### Functional enrichment and GSEA enrichment analyses

DEGs were found between high- and low-risk groups using FDR < 0.05 and |log_2_FC| ≥ 0.585. According to DEGs, the “clusterProfiler” package in R to conduct GO and KEGG enrichment studies and the “ggplot2” tool to create pictures was used, while GSEA 4.2.1 was used to discover related functions and pathway variations in the Hallmark gene set “h.all.v7.4.symbols.gmt.”

### Analysis of tumor microenvironment and immune checkpoints

We started by calculating the scores for 16 immune infiltrating cells and 13 immune-related activities in each sample in both high- and low-risk groups using single-sample Gene Set Enrichment Analysis (ssGSEA) and R package “GSVA.” Then, using the Estimation of STromal and Immune cells in MAlignant Tumor tissues using Expression data (ESTIMATE) program in conjunction with the “estimate” package, the immune-score, stromal-score, and ESTIMATES-score of each sample were determined. Finally, the differences in the scores mentioned above between the two groups were compared. Using the “ggpubr” package, the immune checkpoint activation between high- and low-risk groups was also compared.

The same formula to divide the normalized GEO data into high and low-risk groups based on the median risk score was used. The methods described above are used to externally validate the link between the signature and TIME.

### Mutation analysis

We also obtained the mutation data of THCA patients from the TCGA database (https://portal.gdc.cancer.gov/). The “maftools” package was used to further analyzed the mutation data (Mayakonda et al., 2018). We calculated the tumor mutation burden (TMB) of each sample using the following formula: (total mutation ÷ total covered bases) × 10^6 (Li et al., 2020).

### Cell culture and construction of transfected cell lines

K1 cell line was purchased from the European Collection of Authenticated Cell Culture (ECACC, UK) and K1 cells were maintained in Dulbecco’s modified eagle’s medium (DMEM): Ham’s F12: MCDB 105 (2:1:1) and 2 mM glutamine supplemented with 10% FBS. TPC-1 cell line was purchased from the Chinese Academy of Sciences and TPC1 cells were maintained in DMEM with 15% FBS. All cells were cultured at 37°C, in a humidified atmosphere with 5% CO_2_. Recombinant lentivirus--containing genomes encoding human full-length IPMK, KLF9, SPATA2 (LV-IPMK, LV-KLF9, LV-SPATA2) and a negative control sequence (LV-NC) (GENECHEM, Shanghai, China) were used to generate three types of NRGs-overexpressed cell lines and negative controls (NC). K1 and TPC1 cells were infected with either LV-IPMK or LV-KLF9 or LV-SPATA2 or LV-NC plus 5 μg/ml polybrene (GENECHEM, Shanghai, China). The expression levels of the corresponding genes in each type of gene overexpressing cell line were confirmed by qRT-PCR.

### Quantitative PCR

The First Affiliated Hospital of China Medical University provided 96 pairs of matched tumorous and non-tumorous tissue specimens of PTC. The following table summarizes the clinicopathological characteristics of 96 PTC patients treated at our hospital (Table 2). Total RNA from tissue samples and fresh cultured cells was extracted using RNAiso (Takara, Dalian, China), and then reverse transcribed into cDNA using the QuantiTect Reverse Transcription Kit (Takara, Shiga, Japan). To validate gene expression, quantitative real-time PCR (qRT-PCR) analysis was done using TB-Green (Takara, Shiga, Japan), and the level of GAPDH served as an internal reference. The comparative Ct (2^−ΔΔCt^) method was used to calculate the relative expression. The following primer sequences are available (Table 3).

**TABLE 2:** The clinicopathological features of PTC (*N* = 96).

Clinical variables	Group	Sample (*N* = 96)	Percentage (%)
Age (year), n (%)	≤60	87	90.63
	>60	9	9.37
Gender, n (%)	Female	77	80.21
	Male	19	19.79
Stage, n (%)	Stage I-II	79	82.29
	Stage III-IV	17	17.71
	Unknown	0	0.00
T, n (%)	T1-2	66	68.75
	T3-4	30	31.25
	Tx/unknown	0	0.00
M, n (%)	M0	92	95.83
	M1	4	4.17
	Mx/unknown	0	0.00
N, n (%)	N0	28	29.17
	N1	68	70.83
	Nx/unknown	0	0.00

**TABLE 3:** Premier sequences for qRT-PCR analysis.

Premier	Sequences (5′–3′)
IPMK-F	GTG​CTT​GGC​ATG​AGG​GTT​TAT​C
IPMK-R	TGG​CAG​CAA​CAG​CAT​CTT​TTC
CDKN2A-F	GCG​GAA​GGT​CCC​TCA​GAA​ATG
CDKN2A-R	GCC​AGC​TTG​CGA​TAA​CCA​AA
SPATA2-F	GAC​TTA​TTT​CGG​AAG​TAC​GTG​C
SPATA2-R	GAT​CAG​CCG​GAA​TCG​ATA​AAA​G
KLF9-F	GTG​TCT​GGT​TTC​CAT​TTC​GAA​C
KLF9-R	GAT​CCC​ATA​TCC​TCA​TCT​GGA​C
TNFRSF1B-F	CGG​CTC​AGA​GAA​TAC​TAT​GAC​C
TNFRSF1B-R	ACA​GAA​GAC​TTT​TGC​ATG​TTG​G
FAS-F	GTA​CAC​AGA​CAA​AGC​CCA​TTT​T
FAS-R	TTT​GGT​TTA​CAT​CTG​CAC​TTG​G
AXL-F	AGA​TTT​ATG​ACT​ATC​TGC​GCC​A
AXL-R	TGA​CAT​AGA​GGA​TTT​CGT​CAG​G
GAPDH-F	GTC​TCC​TCT​GAC​TTC​AAC​AGC​G
GAPDH-R	ACC​ACC​CTG​TTG​CTG​TAG​CCA​A

### Cell proliferation assay

Cell proliferation was assessed using the cell counting kit-8 (CCK-8, Dojindo, Japan) assay according to the manufacturer’s protocol. Briefly, 3,000 cells/well were placed into 96-well plate. An aliquot of 10 μl CCK-8 solution was added to each well. After 4 h of incubation at 37°C and 5% CO_2_, the incubation was terminated. Then, the absorbance at 450 nm was measured using a spectrophotometer at 0, 24, 48, and 72 h. For each group, data from five wells were pooled.

### Cell migration and invasion detection

Each group’s cell concentration was adjusted to 5 × 10^4^ cells/ml with serum-free media after 24 h of transfection. The Transwell chamber was filled with 200 μl of cell suspension in the upper chamber and 500 μl of media supplemented with 10% FBS in the lower chamber. The cells were cultured for 12 h for the migration assay and 36 h for the invasion assay, after which they were fixed with 4% paraformaldehyde and stained with 0.5% crystal violet. The invasive cells were then counted in five different visual areas using an inverted microscope at ×100 magnification. Experiments were performed in triplicate.

## Results

### Identification of necroptosis-related DEGs

**FIGURE 1:**
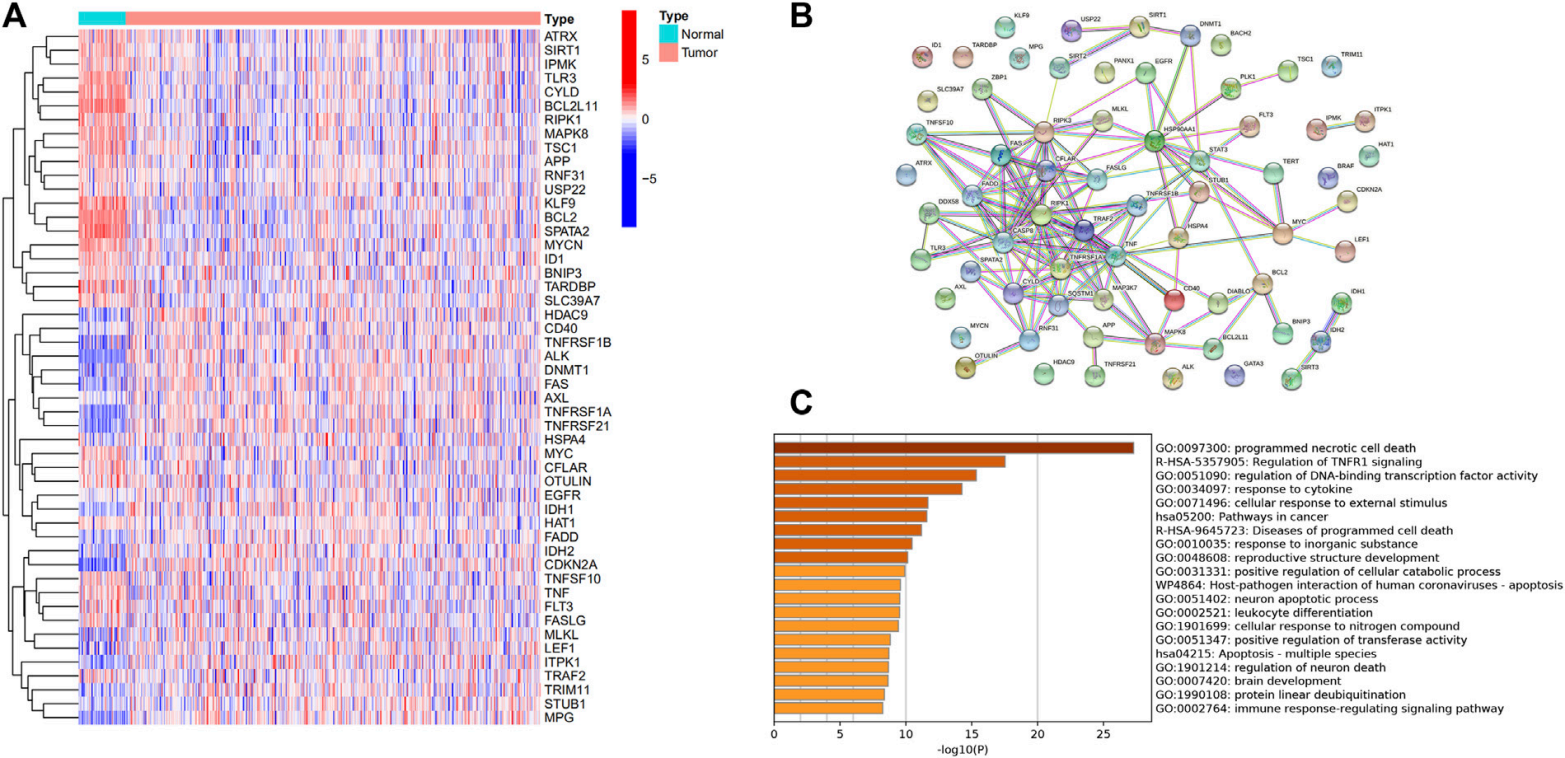
Expression of the necroptosis-related genes in THCA (*N* = 498). **(A)** The heatmap showed the expression levels of 50 necroptosis-related DEGs in normal and tumor samples; **(B)** PPI network indicated the interactions of the necroptosis-related genes; **(C)** GO and KEGG enrichment analysis of necroptosis-related genes.

A total of 50 differentially expressed genes between 510 tumors and 58 adjacent nontumor tissues were identified. Out of 67 necroptosis-related genes, 21 were upregulated and 29 were downregulated in tumors. A heatmap was drawn for showing the 50 genes expression levels (Figure 1A). A PPI network was constructed to show the relationships among all necroptosis-related genes (Figure 1B). GO and KEGG analysis were used to investigate the potential roles of these DEGs, where GO enrichment analysis revealed that these necroptosis-related DEGs are mostly engaged in programmed necrotic cell death, transcription factor activity regulation, and cytokine response. According to KEGG analysis, these genes were primarily involved in cancer and apoptotic pathways (Figure 1C).

### Tumor classification based on DEGs

**FIGURE 2:**
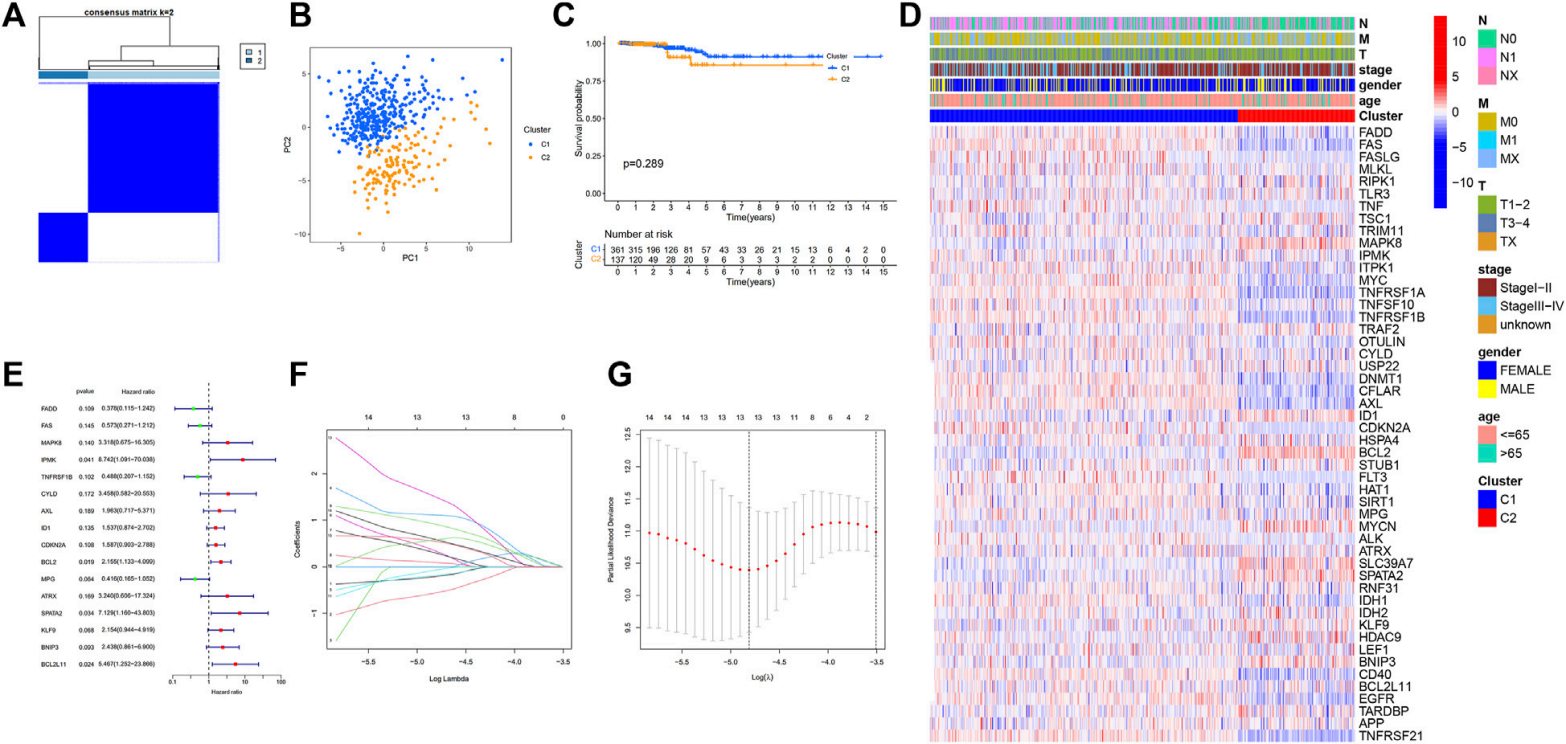
Tumor classification based on the necroptosis-related DEGs; Univariate Cox regression analysis and LASSO analysis. **(A)** 498 PTC patients were grouped into two clusters according to the consensus clustering matrix (*k* = 2); **(B)** Principal Component Analysis (PCA) of RNA expression profile in TCGA cohort; **(C)** Kaplan–Meier curves of overall survival (OS) in two clusters; **(D)** Heatmap and clinicopathologic features of the two clusters; **(E)** Forest plot showing the result of univariate Cox regression analysis of OS, 16 genes with *p* < 0.2; **(F)** Cross-validation for tuning parameter selection in LASSO regression **(G)** LASSO analysis of 16 prognostic pyroptosis-related genes.

To investigate the association between necroptosis-related DEGs and PTC subtypes, consensus clustering analysis on the 50 DEGs using the “ConsensusClusterPlus” program was performed. The letter “k” denoted the number of clusters. When k was adjusted to 2, intragroup correlations were highest while the intergroup correlations were lowest, showing that the 498 PTC patients were classified into two clusters (Figure 2A), C1 (*N* = 361) and C2 (*N* = 137). Additionally, the principal component analysis demonstrated that these patients can be classified into two distinct groups (Figure 2B). However, survival analysis revealed no statistically significant difference in OS between the two clusters (Figure 2C). Following that, a heatmap was created to depict the association between clusters, gene expression profiles, and clinical characteristics (Figure 2D), however, it was discovered that there are minimal clinical distinctions between the two clusters.

### Development of a prognostic gene model and evaluation of predictive effect

**FIGURE 3:**
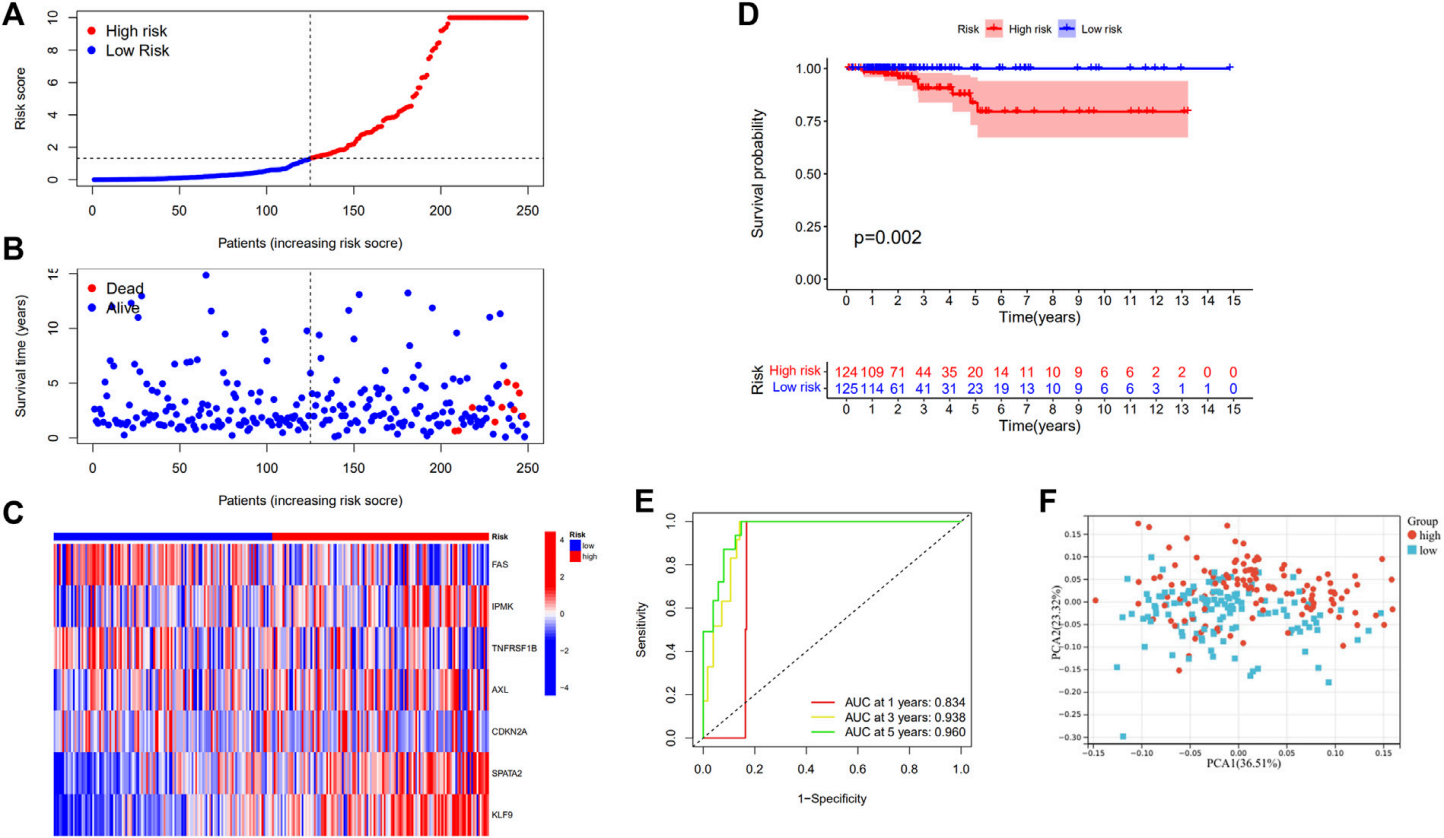
Construction of risk signature in the train set (*N* = 249). **(A)** The distribution of risk score, **(B)** survival status, and **(C)** the expression of seven necroptosis-related genes in high- and low-risk groups; **(D)** Kaplan–Meier curves for OS of THCA patients in high- and low-risk groups; **(E)** Time-dependent ROC analysis, **(F)** PCA of TCGA cohort in the train set.

The models were constructed using the train set. To begin, a univariate Cox regression analysis was used to find survival-related genes. The results indicated that 16 genes in the training set satisfying a threshold with a *p* < 0.2 were associated with survival (Figure 2E). More precisely, 12 genes (MAPK8, IPMK, CYLD, AXL, ID1, CDKN2A, BCL2, ATRX, SPATA2, KLF9, BNIP3, and BCL2L11) were detrimental with HR > 1, whereas four genes (FADD, FAS, TNFRSF1B, and MPG) were protective with HR < 1. The overfitting was reduced by using LASSO Cox regression on the optimum λ value and identified 13 genes (FADD, FAS, IPMK, TNFRSF1B, CYLD, AXL, ID1, CDKN2A, MPG, SPATA2, KLF9, BNIP3, and BCL2L11) that were substantially linked with prognosis (Figures 2F,G). Further study of these genes using multivariate Cox regression analysis resulted in the construction of a prognostic signature consisting of seven necroptosis-related genes. For each sample, a risk score was calculated using the following formula: Risk score = FAS*(−1.172) + IPMK*(2.540) +TNFRSF1B*(−0.975) + AXL*(1.536) + CDKN2A*(1.650) + SPATA2*(4.148) + KLF9*(2.003). The patients were classified in the training set into high- and low-risk groups based on their risk score median value and then ranked and assessed their risk score distributions (Figure 3A). Additionally, the survival status of each sample in the training set was visualized using dot plots and discovered that all deceased patients were concentrated in the high-risk group (Figure 3B). A heatmap was created to depict the differential expression of the seven prognostic genes between the two groups (Figure 3C). Survival analysis revealed that patients with high-risk scores had a significantly worse outcome than those with low-risk scores (*p* = 0.002) (Figure 3D). ROC analysis was utilized to determine the sensitivity and specificity of created prognostic signature, and the areas under the curve (AUC) were found to be 0.834 at 1 year, 0.938 at 3 years, and 0.960 at 5 years, respectively (Figure 3E). The PCA revealed that patients with varying risks were dispersed in two directions (Figure 3F).

Following that, the predictive ability of prognostic signature in the test and total sets was validated. The test and total sets were separated into high- and low-risk groups, respectively, based on the median risk score in the training set. The risk score distribution, survival status, and expression of seven necroptosis-related genes are provided below for each patient in the test set and total set. In both the test and total sets, Kaplan-Meier survival curves indicated that high-risk patients had a shorter OS than low-risk patients. In the test set, the one-year AUC was found to be 0.703, the three-year AUC was 0.656, and the five-year AUC was 0.897, respectively. In the total set, the AUC for the one-year was found to be 0.795, the AUC for the 3 years was 0.822, and the AUC for the 5 years was 0.902. PCA verified the separation of patients into two clusters according to their risk status (Supplementary Figures S2, S3).

### Independent prognostic value of the risk signature

**FIGURE 4:**
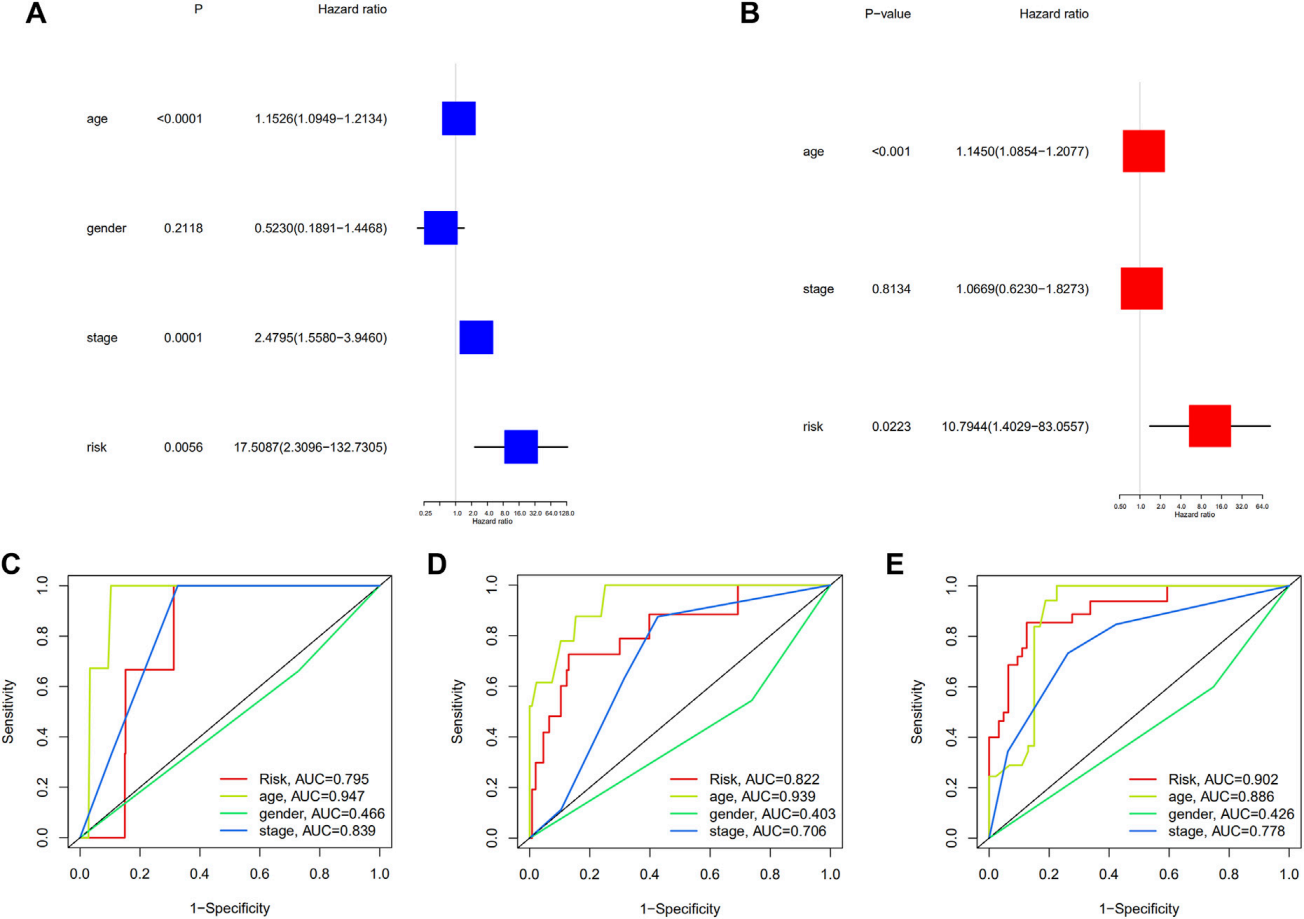
**(A)** Univariate and multivariate, **(B)** Cox regression analyses; The time-dependent ROC to evaluate the prognostic power based on risk score and clinical factors in **(C)** 1-year, **(D)** 3-years, **(E)** 5-years.

The univariate Cox regression analysis revealed that the factors affecting the prognosis were age, clinical stage, and risk grouping (Figure 4A). After adjusting for other confounding factors, it was discovered that age and risk grouping were independent predictors of OS in patients with PTC by multivariate Cox regression analysis (*p* < 0.001, *p* = 0.02223, respectively) (Figure 4B). Furthermore, the ROC curves of risk grouping and other clinicopathological variables showed that risk grouping had a higher prediction level, and its prediction ability increased with time (Figures 4C–E). Furthermore, survival analysis revealed that individuals with high-risk had lower overall survival in all clinical subgroups (Supplementary Figures S4A–L). The clinical significance of these seven genes was investigated by determining the correlations between the expression levels of them and the clinicopathological characteristics of PTC. The expression levels of TNFRSF1B correlated significantly with age (*p* < 0.001) (Supplementary Figure S5A). Compared with that in non-extrathyroidal extension (ETE) cases, FAS expression was upregulated in ETE cases (*p* < 0.01), and SPATA2 expression showed the opposite trend (*p* < 0.001) (Supplementary Figures S5B,C). The expression of CDKN2A positively correlated with T stage (*p* < 0.001) (Supplementary Figure S5D). The expression of SPATA2 was higher in patients without lymph node metastasis (*p* < 0.001) (Supplementary Figure S5E). Patients with multifocal cancer had increased KLF9 expression than with patients unifocal cancer (*p* < 0.01) (Supplementary Figure S5F).

### Functional analyses and gene set enrichment analyses based on the risk signature

**FIGURE 5:**
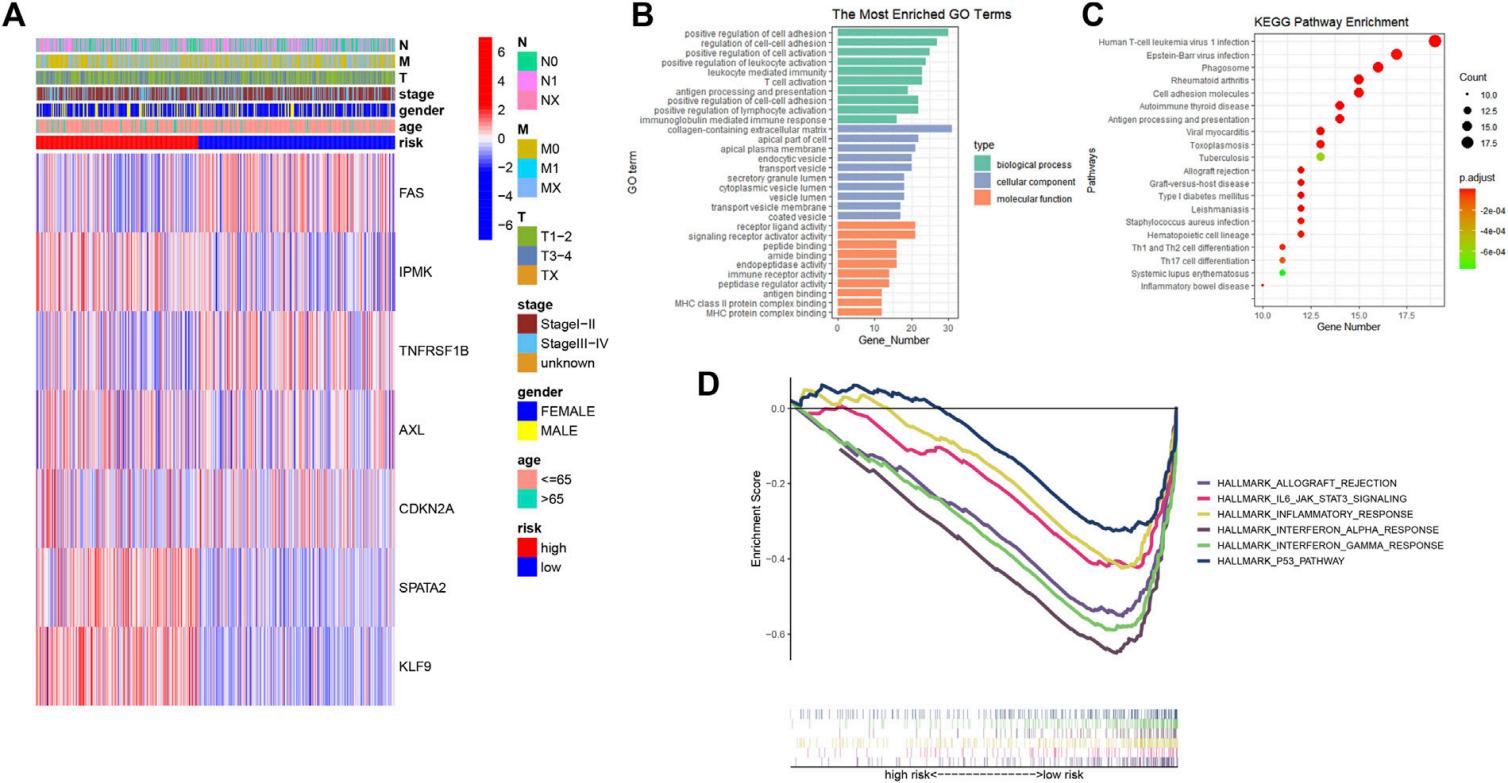
**(A)** Heatmap showing the connections between clinicopathologic factors and high- and low-risk groups; **(B)** Bar chart for GO enrichment of DEGs between high- and low-risk; **(C)** Bubble graph for KEGG pathways of DEGs between high- and low-risk; **(D)** Gene set enrichment analysis (GSEA) showed the significantly enriched hallmarks of tumor sets based on the risk signature in TCGA.

A heatmap was created to depict the link between predictive gene expression and clinical features (Figure 5A). Then, using FDR < 0.05 and |log_2_FC| ≥ 0.585, 268 differentially expressed genes were identified in high- and low-risk groups. Based on DEGs, GO, and KEGG analysis was performed to further elucidate the putative biological activities and pathways associated with the risk signature. According to findings, these DEGs were primarily enriched in cell adhesion, immune-related activities, and several infections or immune-related pathways (Figures 5B,C). The GSEA was used to examine the transcript messages of PTC patients who were grouped by risk score into high- and low-risk categories. In the low-risk group, biological pathways such as interferon-alpha and gamma response, allograft rejection, P53 pathway, inflammatory response, and IL6-JAK-STAT3 signaling were enriched. (Figure 5D).

### Comparison of the immune activity among subgroups

**FIGURE 6:**
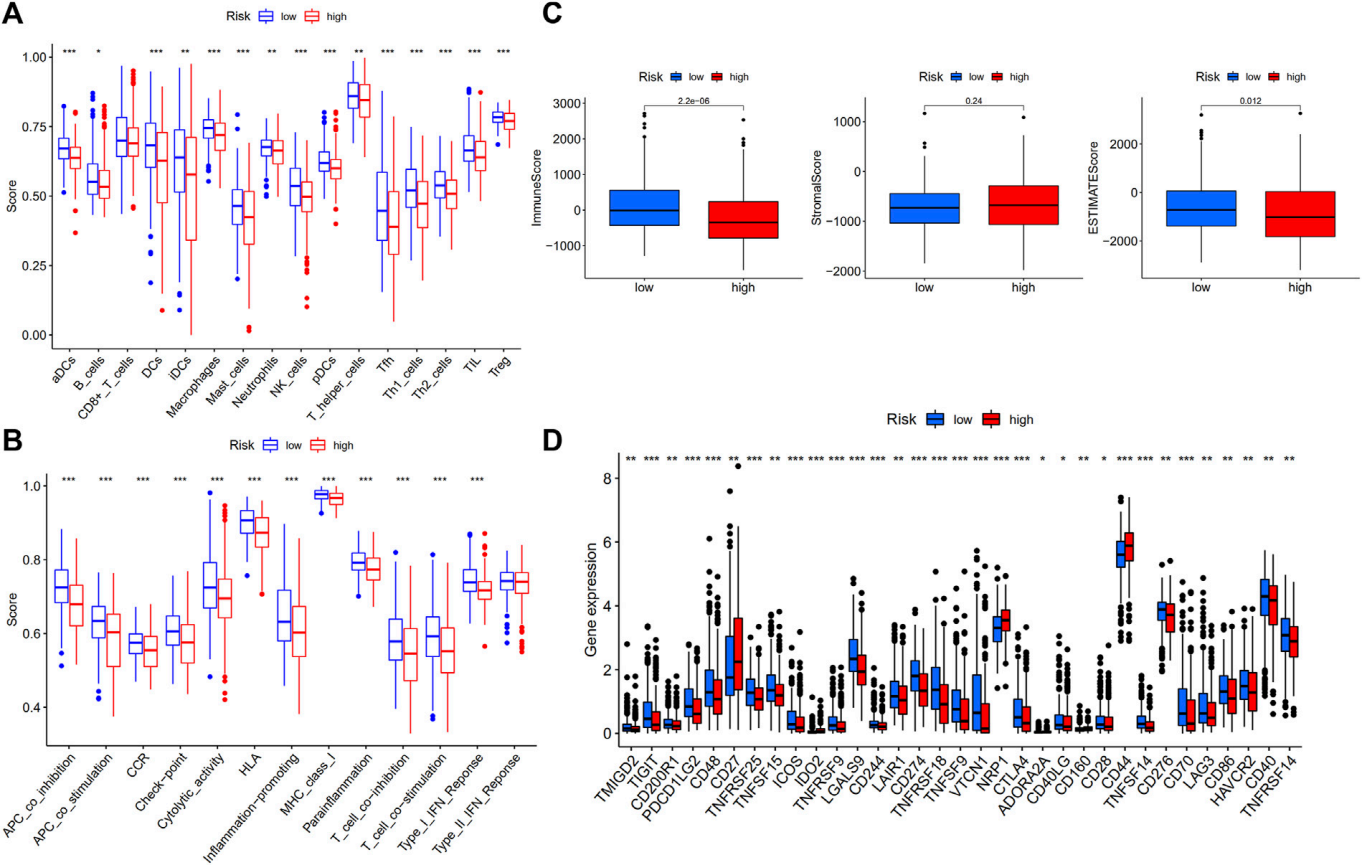
Comparison of ssGSEA scores in high- and low-risk groups in TCGA-THCA database (*N* = 498). **(A)** 16 immune cells and **(B)** 13 immune-related functions between high- and low-risk groups; **(C)** The ESTIMATE scores of high- and low-risk groups; **(D)** Comparison of immune checkpoint expression between high- and low-risk groups.

The ssGSEA was used to compare the enrichment scores of 16 different types of immune cells and the activity of 13 immune-related functions in low- and high-risk groups. The findings revealed that, except for CD8^+^ T cells, which did not differ substantially between the two groups, other immune cells infiltrate at a higher rate in the low-risk subgroup (Figure 6A). Furthermore, the great majority of immune-related pathways were activated more in the low-risk group than in the high-risk group (Figure 6B). We analyzed each immune activity in relation to the risk score in the TCGA samples and found that the higher the risk score, the less active the immune activity was (Supplementary Figures S6, S7). Each sample’s immune-score, stromal-score, and ESTIMATES-score were calculated to determine the estimated amount of stromal and immune cells in the tumor sample. It was observed that the low-risk group contained more immune cells than the high-risk group, but the difference in stromal cell composition between the two groups was not statistically significant. Furthermore, ESTIMATES-score was greater in the low-risk group, indicating reduced tumor purity (Figure 6C). The potential differences in immune checkpoint expression between the two groups were investigated, and the results revealed that most immune checkpoints were more expressed in the low-risk group (Figure 6D). Similar outcomes were reached when analyzing the immunological state of GEO cohort. The low-risk group had more immune infiltration and immune checkpoint expression than the high-risk group (Supplementary Figure S8).

### The correlation between necroptosis-related gene signature and mutation profile

Gene mutation is one of the significant factors in thyroid cancer tumorigenesis and development. We assessed the association between the gene signature and mutation profile in TCGA thyroid cancer samples. We found that the mutation profiles were similar between low- and high-risk groups, and the top three mutated genes were all BRAF, NRAS, and HRAS (Supplementary Figures S9A,B). TMB was not statistically different between high and low risk groups, but was relatively higher in the low-risk group (Supplementary Figure S9C).

### The expression levels of seven prognostic genes

**FIGURE 7:**
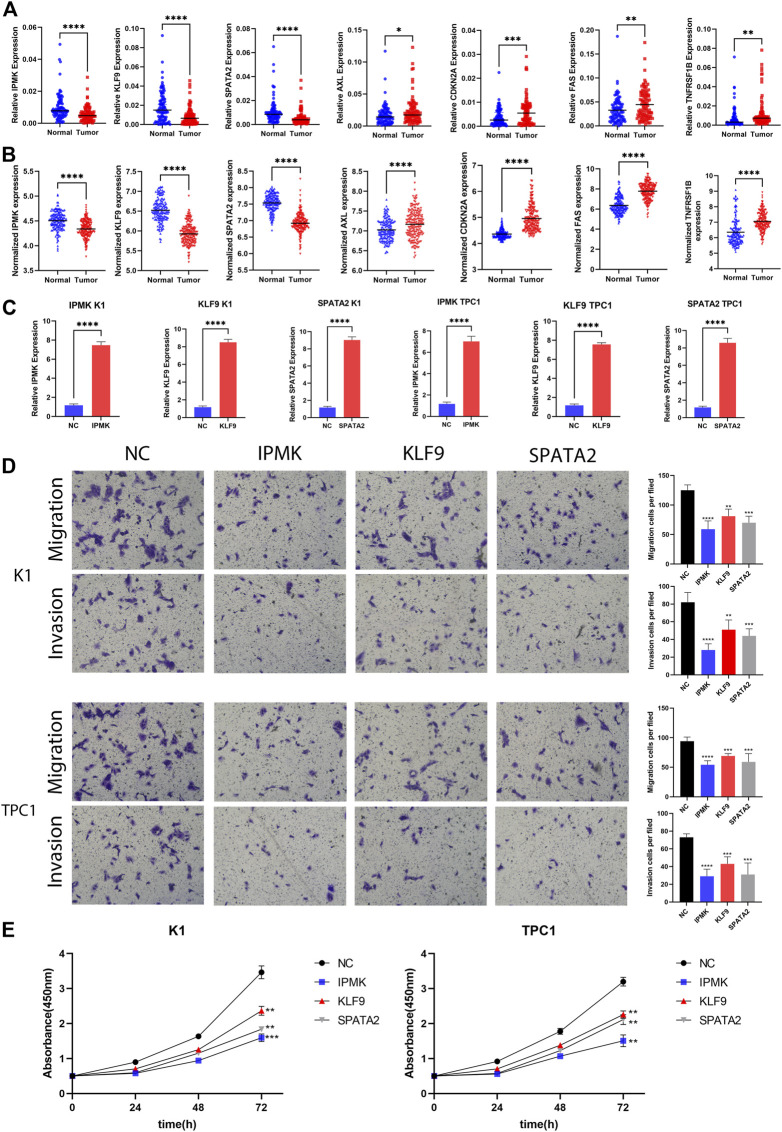
Validation of gene expression and cell function assays. **(A)** The expression levels of IPMK, KLF9, SPATA2, AXL, CDKN2A, FAS, and TNFRSF1B quantified using qRT-PCR analysis in 96 paired thyroid cancer tissues and no-tumorous samples; **(B)** The expression levels of IPMK, KLF9, SPATA2, AXL, CDKN2A, FAS, and TNFRSF1B in GEO; **(C)** Expression level of IPMK, KLF9, and SPATA2 confirmed by qRT-PCR; **(D)** Transwell assays were used to evaluate the migration and invasion in PTC cells after IPMK or KLF9 or SPATA2 overexpression or NC; **(E)** CCK-8 assay was used to evaluate the proliferation after overexpression with the corresponding genes or NC in PTC cells. **p < 0.01; ***p < 0.001; ****p < 0.0001.

Following that, qRT-PCR experiments were performed to validate mRNA expression using 96 pairs of tumor and normal tissues from PTC patients at China Medical University. The qRT-PCR results revealed that AXL (*p* = 0.0308), FAS (*p* = 0.0134), TNFRSF1B (*p* = 0.0250), and CDKN2A (*p* = 0.0224) mRNA expression were considerably greater in tumor tissues. In contrast, tumor samples had lower levels of IPMK (*p* = 0.0010), SPATA2 (*p* = 0.0071), and KLF9 (*p* = 0.0002). The expression of these genes was consistent with TCGA and GEO datasets, which adds to the authenticity and confidence of our developed signature (Figures 7A,B).

### Overexpression of IPMK, KLF9, and SPATA2 significantly inhibit the proliferation, migration, and invasion of PTC cells

We next wanted to further investigate the function of these genes in PTC. By reviewing a large literature, we discovered that the functions of FAS (Basolo et al., 2000; Mitsiades et al., 2006), TNFRSF1B (Lan et al., 2018), AXL (Avilla et al., 2011), and CDKN2A (Ferru et al., 2006; Han et al., 2020; Shi et al., 2021) have already been described in PTC as pro oncogenes, while the functions of IPMK, KLF9, and SPATA2 are unknown in PTC. Therefore, we chose IPMK, KLF9, and SPATA2 as research subjects to demonstrate their roles in PTC cells. We first overexpressed IPMK, KLF9 and SPATA2 using lentiviral vectors in two cell lines, K1 and TPC1, respectively. qRT-PCR assays revealed that IPMK, KLF9, and SPATA2 mRNA expression levels were significantly overexpressed in K1 and TPC1 cells (Figure 7C). Furthermore, the CCK-8 experiment revealed that the IPMK, KLF9, and SPATA2 overexpressed cell lines had lower proliferation ability than control cells (K1-NC and TPC1-NC) (Figure 7E). The transwell assay suggested that overexpression of IPMK, KLF9 and SPATA2 also inhibited cell migration and invasion of K1 and TPC1 cells (Figure 7D).

## Discussion

Thyroid cancer is the most frequent endocrine malignancy, with PTC accounting for more than 85% of all follicular-derived well-differentiated thyroid tumors (Wiltshire et al., 2016). Even though most PTCs are well differentiated, with a low risk of local invasion, recurrence, or metastasis (Cabanillas et al., 2016), some patients with more advanced or aggressive variations frequently exhibit heterogeneity with distinct clinical, pathological, and molecular features (Póvoa et al., 2020; Chen et al., 2021a). According to the most recent American Thyroid Association (ATA) guidelines, these pathological subtypes confer an intermediate risk of recurrence (Haugen et al., 2016), and these variants are associated with higher rates of recurrence and metastasis, as well as, in some cases, minimal efficacy of radioiodine therapy and may have inferior survival (Lam et al., 2005). Due to a lack of understanding of the natural history of these more aggressive variants, treatment for these patients is frequently inadequate or suboptimal. Therefore, carrying out risk stratification management is critical for further optimizing the diagnosis and treatment of PTC patients.

Necroptosis is a type of programmed cell death that can address apoptosis resistance while also activating and enhancing antitumor immunity in cancer treatment (Kowalski et al., 2017), which can be triggered by cytokines, danger signals, or pathogen infection (Linkermann and Green, 2014). After being induced, complex upstream signals eventually converge on the activation of RIPK3, which binds and directly phosphorylates MLKL, a crucial effector of necroptosis and the pathway’s most downstream component known to be necessary (Zhao et al., 2012). In contrast to apoptosis, this type of cell death is distinguished by plasma membrane permeabilization and the production of damage-associated molecular patterns (DAMPs), which have significant immunological effects (Pasparakis and Vandenabeele, 2015). Necroptotic cells may supply antigens and inflammatory cytokines to dendritic cells (DCs) for antigen cross-priming, which activates cytotoxic CD8^+^ T lymphocytes, resulting in tumor cell eradication through the release of DAMPs into the tissue milieu (Gong et al., 2019). However, necroptotic cells may recruit immune inflammatory cells and induce inflammation, which can promote tumor development by boosting angiogenesis, cancer cell proliferation, and metastasis. A study showed that human and murine tumor cells stimulate endothelial cells to undergo programmed necrosis (necroptosis), which facilitates tumor cell extravasation and metastasis. RIPK1 inhibitor necrostatin-1 or endothelial-cell-specific RIPK3 deletion inhibited tumor cell-induced endothelial necroptosis, tumor cell extravasation, and metastasis in mice (Strilic et al., 2016). As can be observed, the role of necrotic apoptosis in tumors is extremely complex. The potential significance of necroptosis-related genes in PTC, on the other hand, is uncertain. As a result, an attempt was made to identify potential diagnostic markers of necroptosis using targeted immunotherapy to increase the survival of PTC patients and to investigate the prognostic and diagnostic relevance of necroptosis. This study’s findings imply that inducing necroptosis with immunotherapy may be a viable treatment strategy for improving patient outcomes.

In this work, we looked at the expression of 67 necroptosis-related genes in PTC samples and adjacent samples and discovered that 50 of them were expressed differently, where 21 of these genes were upregulated, and 29 were downregulated. A consistent cluster analysis on PTC samples was conducted based on NRG expression and discovered that PTC samples can be separated into two subgroups, implying that NRGs may be involved in PTC subclassification. Following that, seven genes related to prognosis (IPMK, AXL, CDKN2A, SPATA2, KLF9, FAS, and TNFRSF1B) were obtained and constructed a seven genes prognostic risk signature from NRGs using a series of processes such as univariate Cox regression analysis, LASSO regression analysis, and multivariate Cox regression analysis. Inositol polyphosphate multikinase (IPMK) is a conserved enzyme that belongs to the inositol phosphokinase 6-kinase family and plays a key role in the phosphorylation of inositol phosphates (IPs) (Irvine and Schell, 2001; Otto et al., 2007). During necroptosis, IPMK is also required for activated phospho-MLKL to oligomerize and relocate to the plasma membrane. IPMK, in particular, may generate highly phosphorylated IPs in cells from lowly phosphorylated precursors, and highly phosphorylated IPs can influence necroptosis by directly binding MLKL and controlling its function (Dovey et al., 2018). It was discovered that IPMK expression was lower in PTC tumor tissues than in normal tissues, but that high expression was linked with a poor prognosis in PTC. AXL is a tyrosine kinase receptor that belongs to TAM (TYRO3, AXL, and MER) receptor family. TAM family regulates cell growth, survival, and proliferation (Lemke and Rothlin, 2008; Lemke, 2013). AXL is frequently overexpressed in cancer, which can increase the invasiveness and motility of the normally non-invasive MCF-7 cell line (Zhang et al., 2008). AXL has been demonstrated to boost the motility, invasion, proliferation, and survival of breast cancer cells (Gjerdrum et al., 2010). Importantly, a study evaluating transcriptome analysis of 941 cancer cell lines found that high AXL mRNA levels indicate resistance to necroptosis while low RIPK3 mRNA levels predict resistance to necroptosis (Najafov et al., 2018). AXL behaved as a cancer-promoting gene in our sample validation due to its overexpression in tumor tissues and its negative connection with survival time. Although cyclin-dependent kinase inhibitor 2A (CDKN2A) is frequently altered or deleted in a wide range of cancers and is known to be an important tumor suppressor gene, a study suggested that CDKN2A plays a key role in the formation and progression of larynx squamous cell carcinoma (Sasiadek et al., 2004). Furthermore, CDKN2A hypermethylation may be a risk factor for a poor prognosis of pancreatic cancer (Xing et al., 2013). CDKN2A expression differed considerably between tumor and normal tissues in our investigation. It was more abundant in tumors, and increased expression in malignancies is associated with a poor prognosis. A genome-wide siRNA screen identified Spermatogenesis-associated 2 (SPATA2) as a gene implicated in necroptosis mediation (Hitomi et al., 2008). SPATA2 modulated RIPK1 activation *via* altering its M1 ubiquitination. More particular, SPATA2 deficiency can hasten the M1 ubiquitination of RIPK1, resulting in necroptosis resistance (Wei et al., 2017). According to research, increased SPATA2 expression is related to a poor prognosis in various cancers, such as ovarian or cervical cancer (Wieser et al., 2019; Wieser et al., 2020). We found high SPATA2 expression in tumor tissues from patients with a relatively poor prognosis. Kruppel-like factor 9 (KLF9), also known as basic transcription element-binding protein 1, is a transcriptional regulator involved in cellular adhesion, differentiation, and proliferation. It is reported to be downregulated in various cancers, including endometrial carcinoma and colorectal cancer (Kang et al., 2008; Simmen et al., 2008). Sadia et al. discovered that KLF9 expression was significantly lower in cervical cancer patients compared to healthy controls and that it was significantly lower in the advanced tumor stage and distant metastatic groups compared to the lower tumor stage and non-metastatic groups (Safi et al., 2021). However, Chen et al. (2021b) discovered that KLF9 expression was positively associated with acute myeloid leukemia. Consequently, KLF9 may have a distinct role in various cancer types. KLF9 appeared to be a cancer suppressor gene in our investigation, as it was downregulated threefold in tumor tissues; yet, it also contributed to patient survival shortening since it was enriched in the high-risk group. Fas cell surface death receptor (FAS) has been found to play a critical role in the physiological regulation of programmed cell death as a member of TNF-receptor superfamily and has been implicated in the pathogenesis of several malignancies and immune system diseases (Vandenabeele et al., 2010). FAS mRNA and protein expression levels were considerably lowered in breast carcinoma, whereas high FAS expression implies a better prognosis in breast cancer patients (Zhang et al., 2021). FAS was demonstrated to be increased in tumor samples in our investigation, and its high expression in PTC patients predicted better survival, implying that FAS may be a tumor suppressor gene. Tumor necrosis factor receptor-2 (TNFR2/TNFRSF1B) is a cell-surface receptor that regulates cell survival and proliferation and is abundantly expressed on the surface of many human tumors (Chen et al., 2007). Recently, the possibility of targeting this receptor as a next-generation cancer therapy method has emerged (Chen and Oppenheim, 2017; Vanamee and Faustman, 2017). It was discovered that the gene was more abundant in tumor tissues and that increased expression was associated with a better prognosis. We performed cellular functional assays on three genes not previously reported to function in PTC, and we confirmed the overexpression of IPMK, KLF9, or SPATA2 could inhibit the proliferation, migration, and invasion of PTC cells.

The risk score for each PTC patient was estimated using the risk signature and classified into high- and low-risk groups based on the median value of all patient risk scores. Kaplan–Meier survival curves revealed that high-risk patients had a shorter OS than low-risk individuals. Additionally, the AUC of the receiver operating characteristic curve demonstrated that the risk signature was effective at predicting survival prognosis. Cox regression analysis, both univariate and multivariate, established that the created risk signature was an independent risk factor for prognosis. Through the above analysis, it was proved that the signature has a good ability to predict prognosis. Functional enrichment analysis was also performed on differentially expressed genes across high- and low-risk groups and discovered that these genes were primarily involved in cell adhesion, immunological function, and pathogen infection. GSEA revealed a high enrichment of immune-related pathways in the low-risk categories. As a result, it is hypothesized that necroptosis may be associated with the immunological microenvironment of PTC. The calculated scores for immune cell infiltration and immunological function in high- and low-risk groups using ssGSEA revealed that the patients in the low-risk group had a higher total immunological activity. Additionally, ESTIMATE algorithm was employed to estimate the tumor purity of each sample. To be more precise, the greater the immune- and stromal-scores, the less pure the tumor. This indicated that the high-risk group has a higher tumor purity, which is frequently associated with a poor prognosis (Yoshihara et al., 2013). Cancer immunotherapy has experienced remarkable advances in recent years and more and more potential immune checkpoints have been found (Charoentong et al., 2017; van den Bulk et al., 2018). As a result, the degree of expression of common immune checkpoint proteins in high- and low-risk groups was also investigated, which revealed that the great majority of immune checkpoint proteins were expressed at a higher level in the low-risk group, implying that immunotherapy may be more successful for low-risk patients. TMB can be used to predict the ability to respond to immunotherapy, with higher TMB representing a higher likelihood of being immunotherapy effective (Jardim et al., 2021). Although there was no statistical difference in TMB between low- and high-risk groups, the relatively high TMB of low-risk group represented its better response to immunotherapy, which was consistent with our previous conclusion.

Since there are few studies on the involvement of necroptosis in thyroid cancer at the moment, this study gives some theoretical foundations and research ideas for the way forward. However, this research encountered certain limitations. To begin, due to a lack of sample survival data in GEO database, the prediction ability of the signature to prognosis is only internally validated in TCGA database, and hence were unable to obtain sufficient data from other sources to externally validate the signature-prognosis association. Furthermore, additional fundamental investigations are required to study the relationship between the signature and the tumor microenvironment. As a result, additional research is required to substantiate the above-mentioned conclusion.

## Conclusion

In general, this study revealed the expression and prognostic value of NRGs in PTC and developed a risk prediction signature of necroptosis-related genes. The created signature contributes to prognostic prediction as well as immunotherapy evaluation in PTC patients. These results may provide ideas for further investigation of the role of necroptosis in PTC and guide clinicians towards individualized treatments for PTC patients with different clinical characteristics.

## Data Availability

Publicly available datasets were analyzed in this study. This data can be found here: Gene expression profiles and clinical information of THCA and gene expression profiles of GEO in this study are available from the public database (TCGA, https://portal.gdc.cancer.gov/; GEO, https://www.ncbi.nlm.nih.gov/geo/).
