# Supplementary figures and images for: A novel necroptosis-related gene signature associated with immune landscape for predicting the prognosis of papillary thyroid cancer
Source: Front Genet. 2022 Sep 15;13:947216. doi: 10.3389/fgene.2022.947216 (PMC9520455; doi:10.3389/fgene.2022.947216)

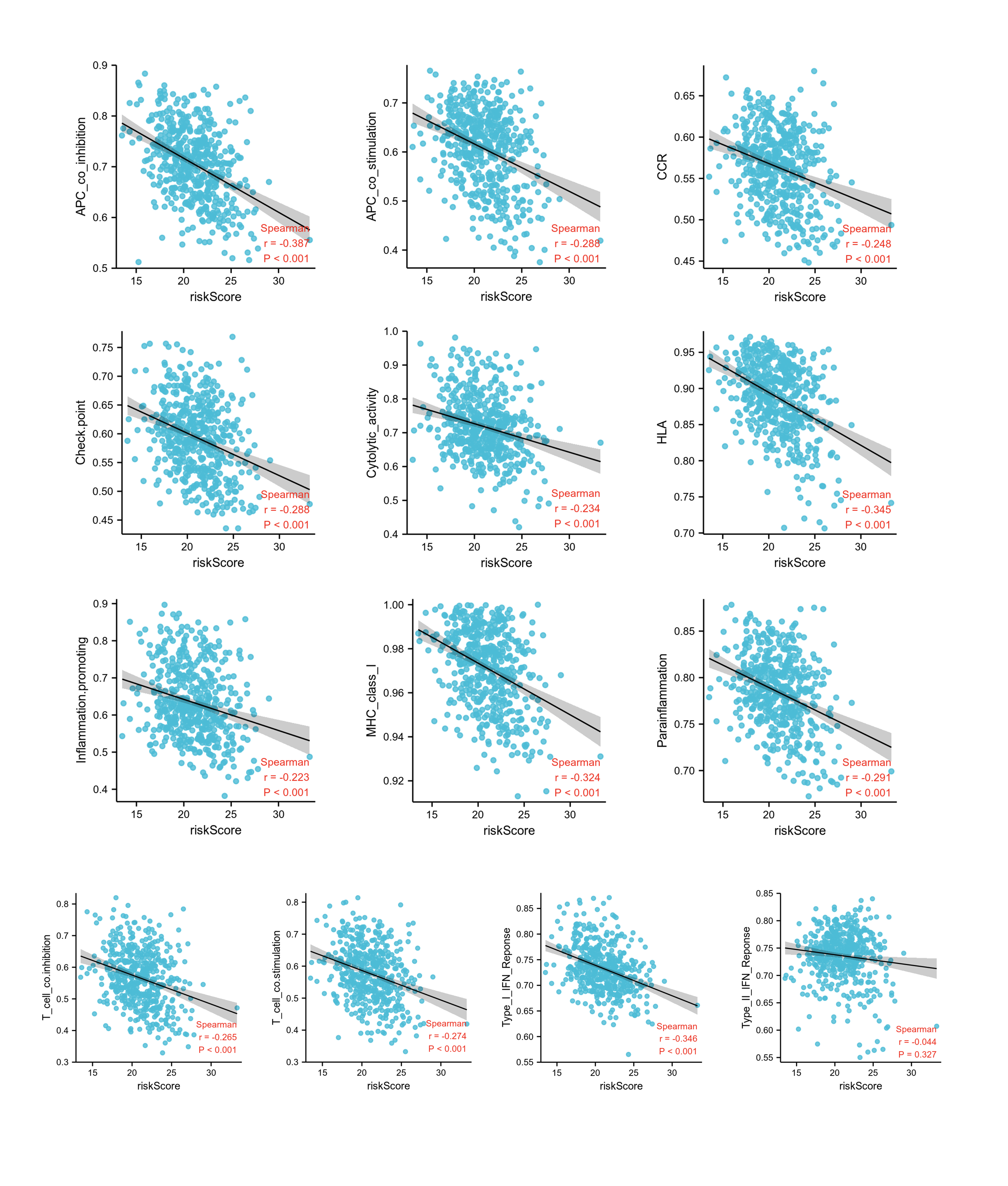

Supplement: Supplementary file 1 [file Image6.TIF]

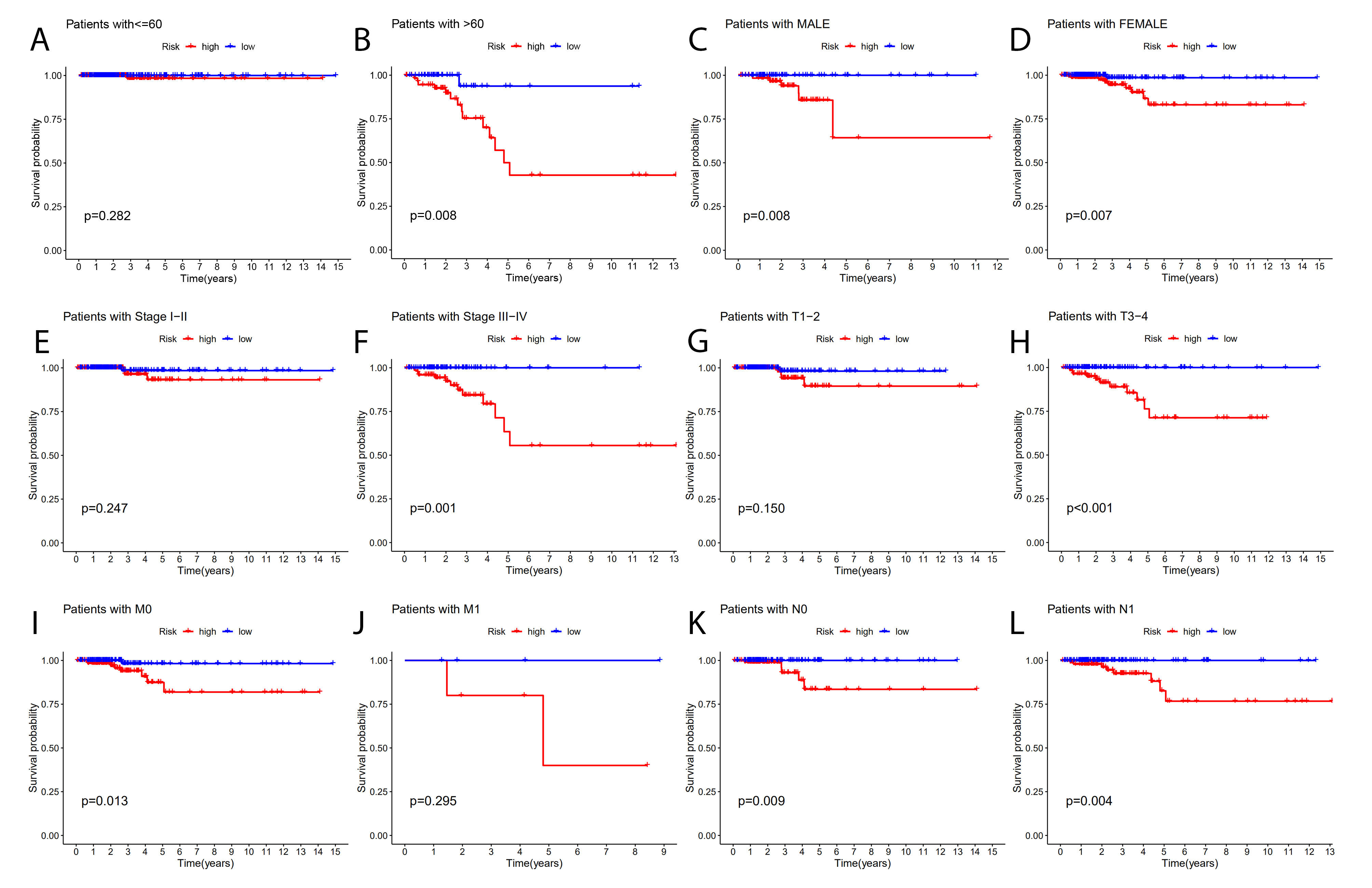

Supplement: Supplementary file 3 [file Image4.TIF]

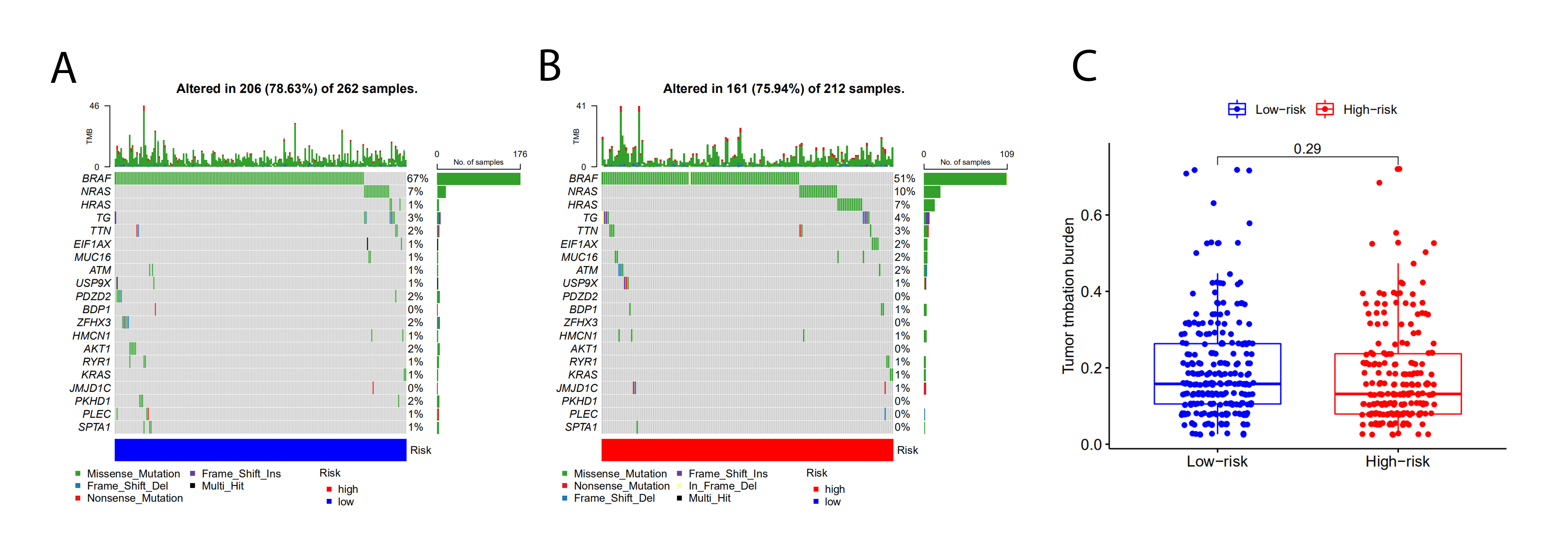

Supplement: Supplementary file 4 [file Image9.TIF]

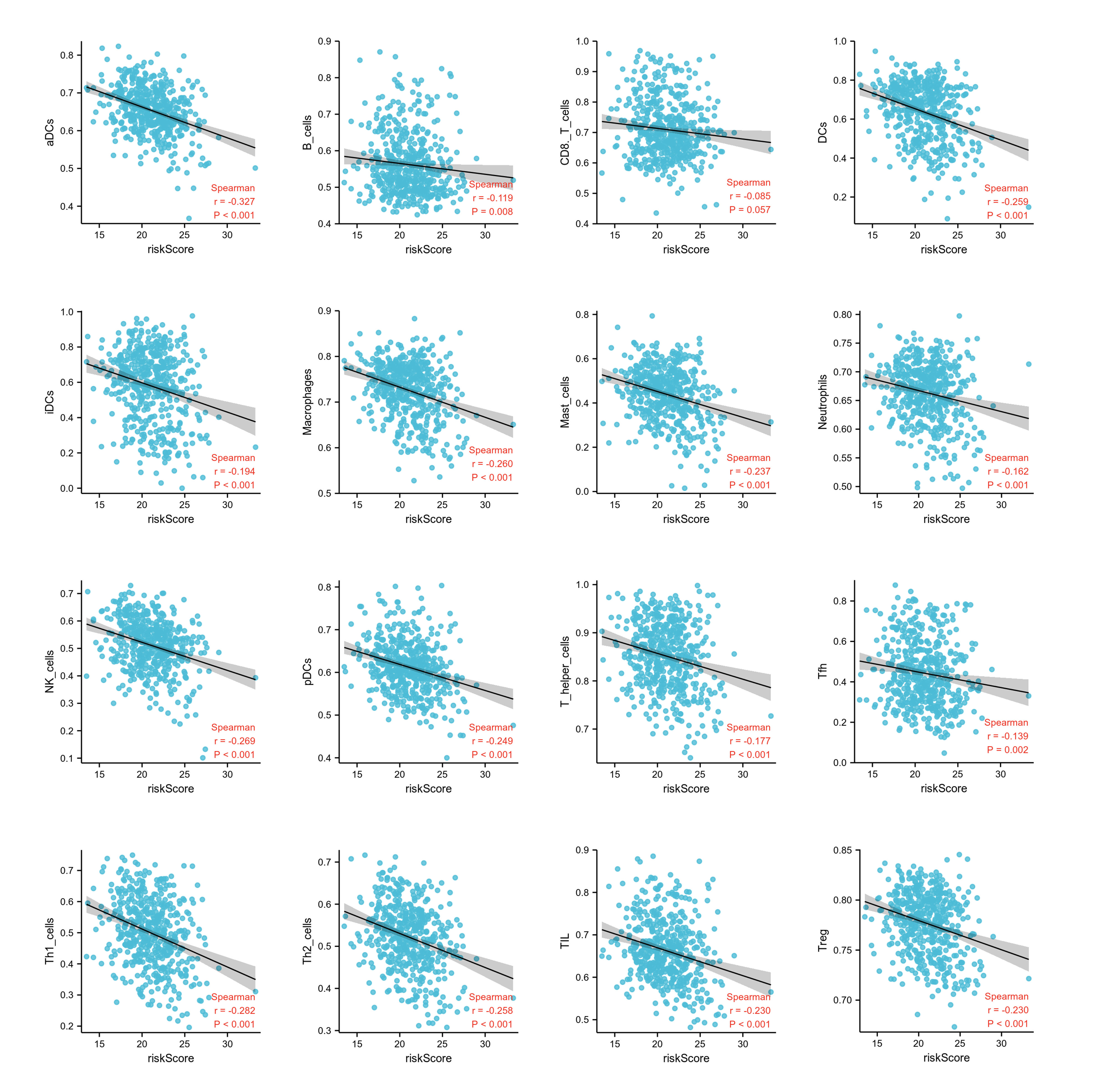

Supplement: Supplementary file 5 [file Image7.TIF]

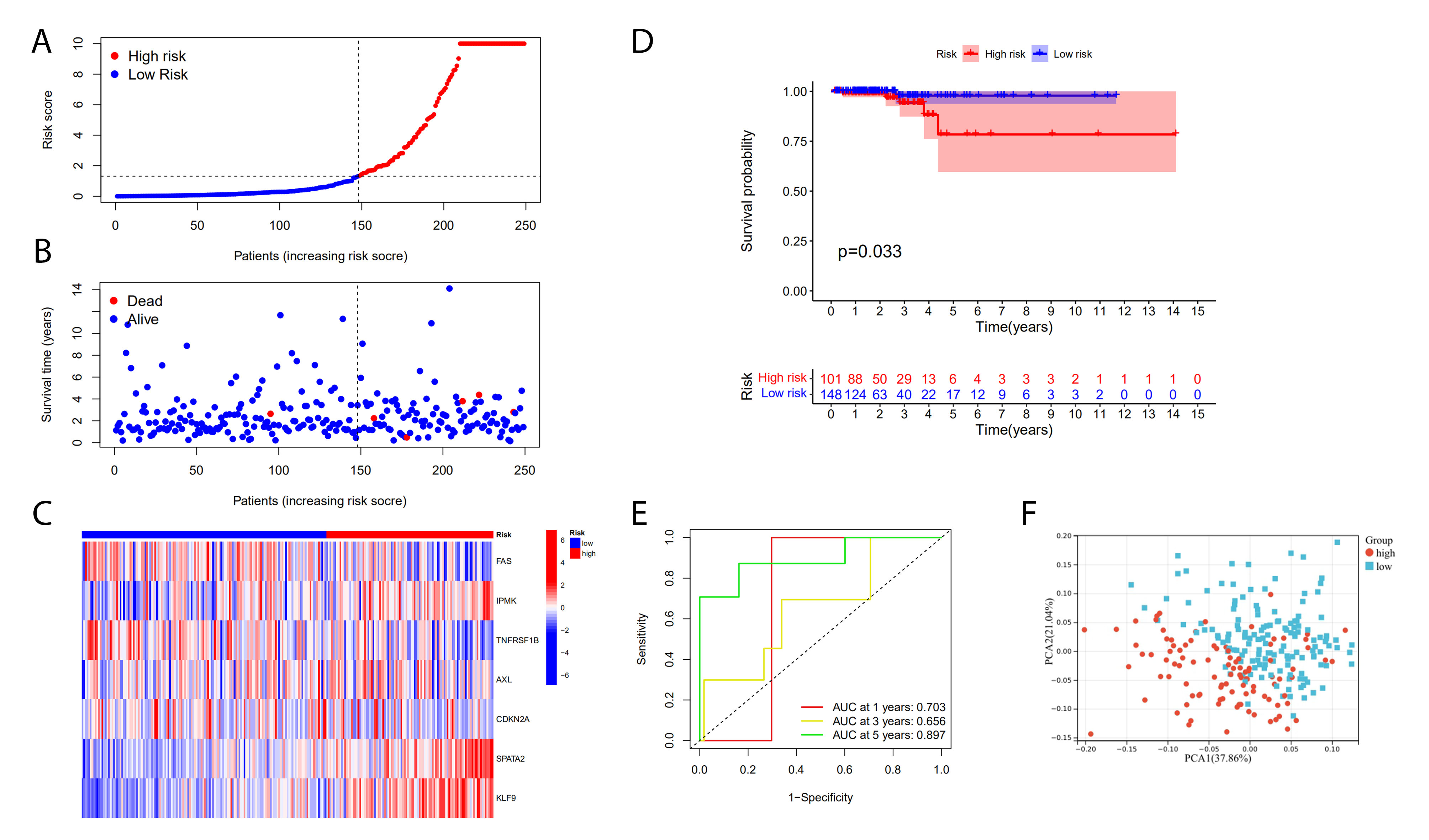

Supplement: Supplementary file 8 [file Image2.PNG]

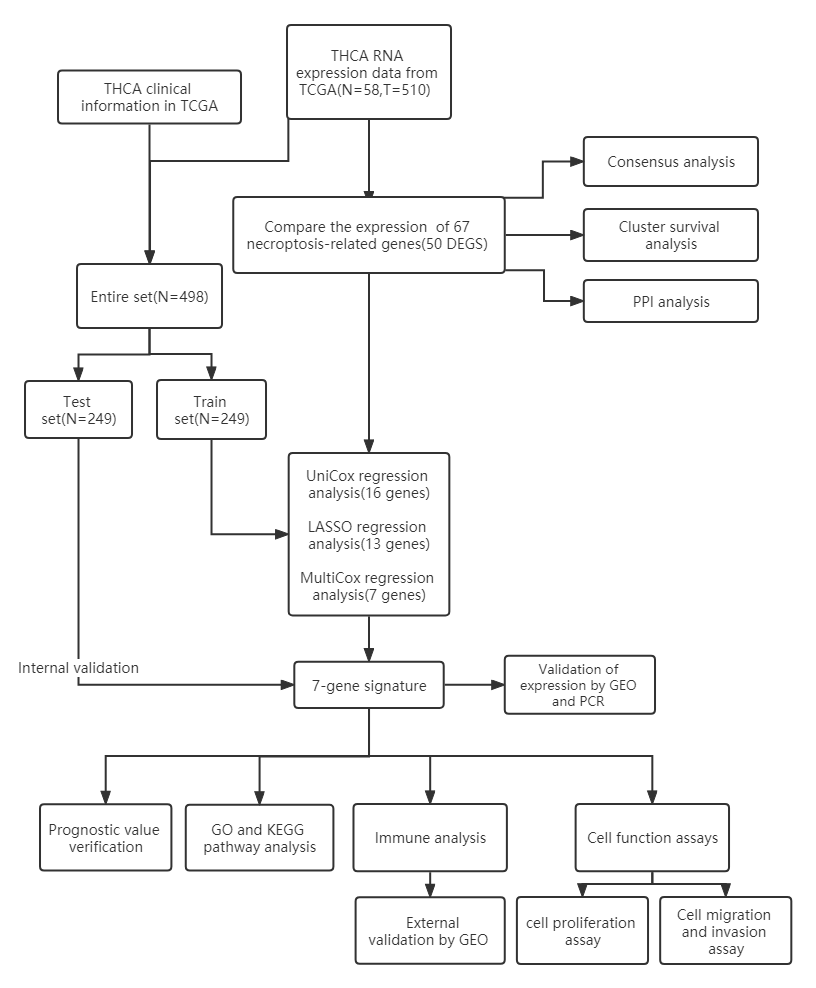

Supplement: Supplementary file 10 [file Image1.PNG]

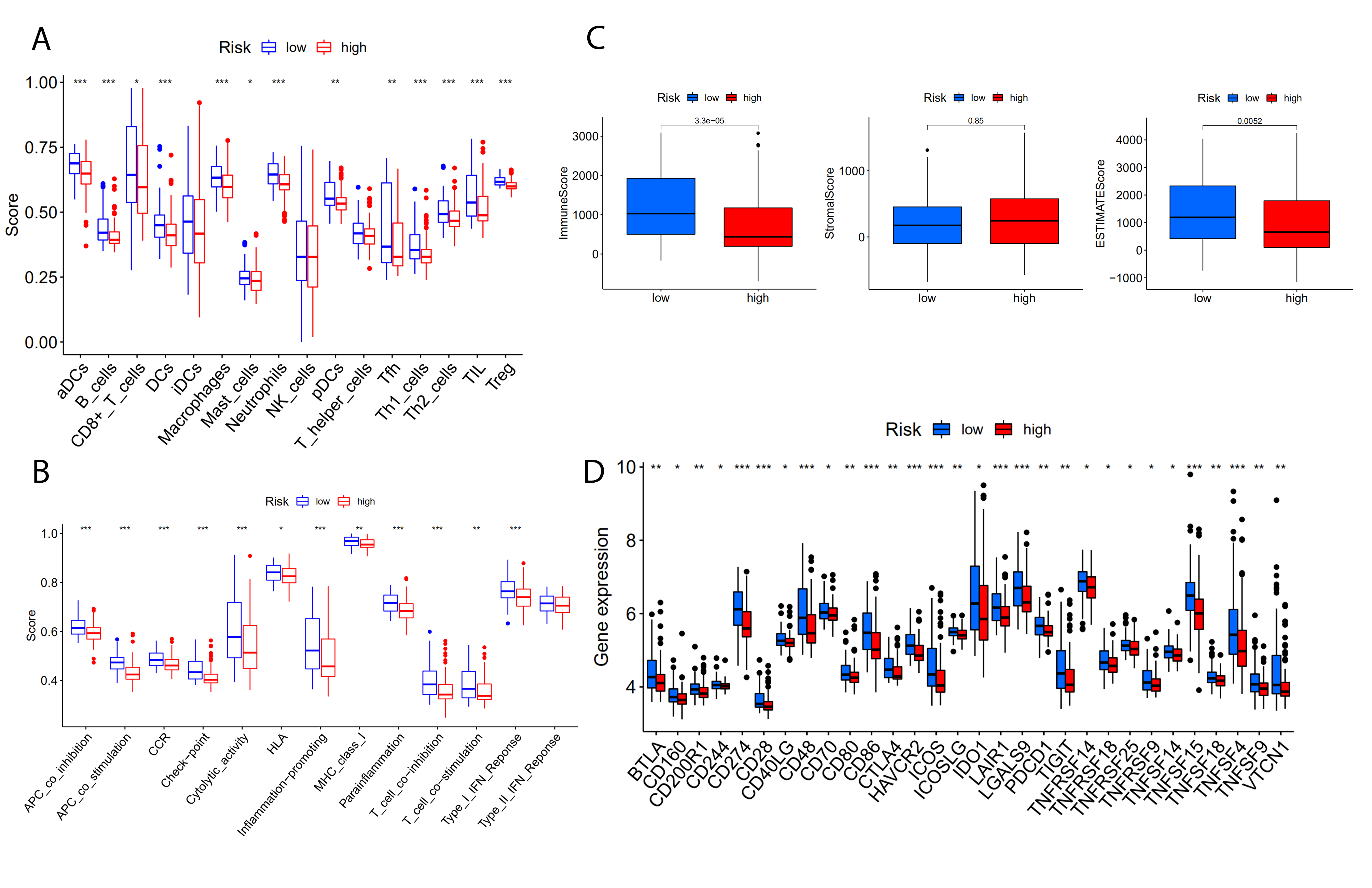

Supplement: Supplementary file 11 [file Image8.TIF]

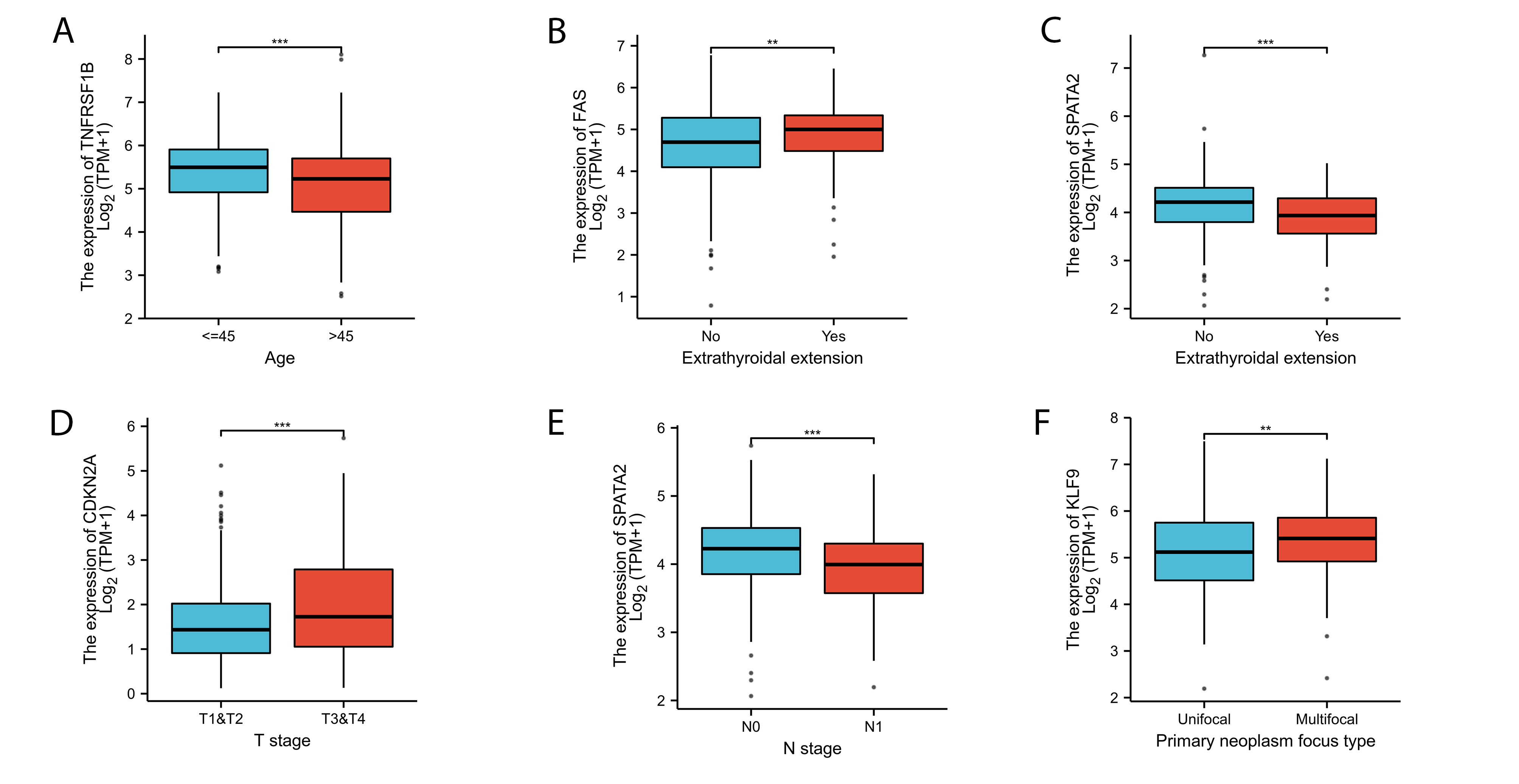

Supplement: Supplementary file 12 [file Image5.TIF]

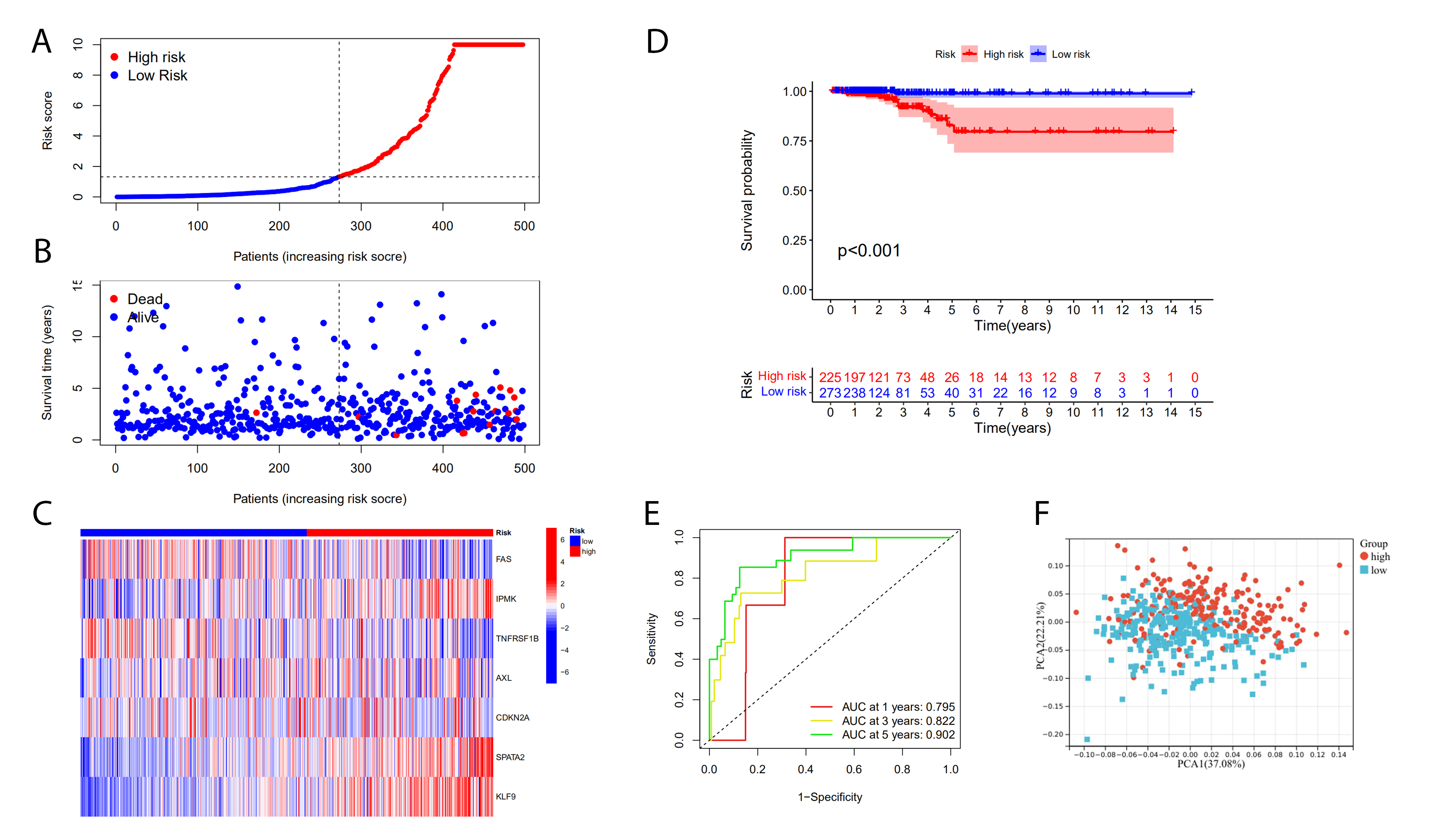

Supplement: Supplementary file 13 [file Image3.PNG]
