# Supplementary material for: A novel necroptosis-related gene signature associated with immune landscape for predicting the prognosis of papillary thyroid cancer
Source: Front Genet. 2022 Sep 15;13:947216. doi: 10.3389/fgene.2022.947216 (PMC9520455; doi:10.3389/fgene.2022.947216)
Supplement: Supplementary file 6 [file Table2.DOCX]

Table1. The clinical characteristics in training, test and total sets

| Variables | Group | Training set(N=249) | Test set(N=249) | Total set(N=498) | P value |
| --- | --- | --- | --- | --- | --- |
| Age(year) | ≤60 | 196 (78.71%) | 190 (76.31%) | 386 (77.51%) | 0.520 |
|  | ＞60 | 53 (21.29%) | 59 (23.69%) | 112 (22.49%) |  |
| Gender, n (%) | Male | 62 (24.90%) | 73 (29.32%) | 135 (27.11%) | 0.267 |
|  | Female | 187 (75.10%) | 176 (70.68%) | 363 (72.89%) |  |
| Stage, n (%) | Stage I-II | 164 (65.86%) | 167 (67.07%) | 331 (66.47%) | 0.362 |
|  | Stage III-IV | 83 (33.33%) | 82 (32.93%) | 165 (33.13%) |  |
|  | Unknow | 2 (0.80%) | 0 (0.00%) | 2 (0.40%) |  |
| T, n (%) | T1-2 | 143 (57.43%) | 161 (64.66%) | 304 (61.04%) | 0.252 |
|  | T3-4 | 105 (42.17%) | 87 (34.94%) | 192 (38.55%) |  |
|  | Tx/unknow | 1 (0.40%) | 1 (0.40%) | 2 (0.40%) |  |
| M, n (%) | M0 | 152 (61.04%) | 130 (52.21%) | 282 (56.63%) | 0.112 |
|  | M1 | 5 (2.01%) | 4 (1.61%) | 9 (1.81%) |  |
|  | Mx/unknow | 92 (36.95%) | 115 (46.18%) | 207 (41.57%) |  |
| N, n (%) | N0 | 105 (42.17%) | 123 (49.40%) | 228 (45.78%) | 0.206 |
|  | N1 | 115 (46.18%) | 105 (42.17%) | 220 (44.18%) |  |
|  | Nx/unknow | 29 (11.65%) | 21 (8.43%) | 50 (10.04%) |  |
