# Supplementary material for: A novel necroptosis-related gene signature associated with immune landscape for predicting the prognosis of papillary thyroid cancer
Source: Front Genet. 2022 Sep 15;13:947216. doi: 10.3389/fgene.2022.947216 (PMC9520455; doi:10.3389/fgene.2022.947216)
Supplement: Supplementary file 7 [file Table3.DOCX]

Table 2. The clinicopathological features of PTC (N=96)

| Clinical variables | Group | Sample (N=96) | Percentage (%) |
| --- | --- | --- | --- |
| Age(year), n (%) | <=60 | 87 | 90.63% |
|  | >60 | 9 | 9.37% |
| Gender, n (%) | Female | 77 | 80.21% |
|  | Male | 19 | 19.79% |
| Stage, n (%)  T, n (%)  M, n (%) | Stage I-II  Stage III-IV  Unknow  T1-2  T3-4  Tx/unknow  M0  M1  Mx/unknow | 79  17  0  66  30  0  92  4  0 | 82.29%  17.71%  0.00%  68.75%  31.25%  0.00%  95.83%  4.17%  0.00% |
| N, n (%) | N0 | 28 | 29.17% |
|  | N1 | 68 | 70.83% |
|  | Nx/unknow | 0 | 0.00% |
