# Supplementary material for: A novel necroptosis-related gene signature associated with immune landscape for predicting the prognosis of papillary thyroid cancer
Source: Front Genet. 2022 Sep 15;13:947216. doi: 10.3389/fgene.2022.947216 (PMC9520455; doi:10.3389/fgene.2022.947216)
Supplement: Supplementary file 9 [file Table4.DOCX]

Table 3. Premier sequences for qRT-PCR analysis

| **Premier** | **Sequences** **(5**′**–3**′**)** |
| --- | --- |
| IPMK-F | GTGCTTGGCATGAGGGTTTATC |
| IPMK-R | TGGCAGCAACAGCATCTTTTC |
| CDKN2A-F | GCGGAAGGTCCCTCAGAAATG |
| CDKN2A-R | GCCAGCTTGCGATAACCAAA |
| SPATA2-F | GACTTATTTCGGAAGTACGTGC |
| SPATA2-R | GATCAGCCGGAATCGATAAAAG |
| KLF9-F | GTGTCTGGTTTCCATTTCGAAC |
| KLF9-R | GATCCCATATCCTCATCTGGAC |
| TNFRSF1B-F | CGGCTCAGAGAATACTATGACC |
| TNFRSF1B-R | ACAGAAGACTTTTGCATGTTGG |
| FAS-F | GTACACAGACAAAGCCCATTTT |
| FAS-R | TTTGGTTTACATCTGCACTTGG |
| AXL-F | AGATTTATGACTATCTGCGCCA |
| AXL-R | TGACATAGAGGATTTCGTCAGG |
| GAPDH-F | GTCTCCTCTGACTTCAACAGCG |
| GAPDH-R | ACCACCCTGTTGCTGTAGCCAA |
